# Expression of the Flp proteins by *Haemophilus ducreyi *is necessary for virulence in human volunteers

**DOI:** 10.1186/1471-2180-11-208

**Published:** 2011-09-22

**Authors:** Diane M Janowicz, Sean A Cooney, Jessica Walsh, Beth Baker, Barry P Katz, Kate R Fortney, Beth W Zwickl, Sheila Ellinger, Robert S Munson

**Affiliations:** 1 Department of Medicine, Indiana University, School of Medicine, Indianapolis, IN 46202, USA; 2 Department of Microbiology and Immunology, Indiana University, School of Medicine, Indianapolis, IN 46202, USA; 3 The Research Institute at Nationwide Children's Hospital, The Ohio State University, Columbus, Ohio 43205, USA; 4 Department of Pediatrics, The Ohio State University, Columbus, Ohio 43205, USA

## Abstract

**Background:**

*Haemophilus ducreyi*, the causative agent of the sexually transmitted disease chancroid, contains a *flp *(fimbria like protein) operon that encodes proteins predicted to contribute to adherence and pathogenesis. *H. ducreyi *mutants that lack expression of Flp1 and Flp2 or TadA, which has homology to NTPases of type IV secretion systems, have decreased abilities to attach to and form microcolonies on human foreskin fibroblasts (HFF). A *tadA *mutant is attenuated in its ability to cause disease in human volunteers and in the temperature dependent rabbit model, but a *flp1flp2 *mutant is virulent in rabbits. Whether a *flp *deletion mutant would cause disease in humans is not clear.

**Results:**

We constructed 35000HPΔ*flp1-3*, a deletion mutant that lacks expression of all three Flp proteins but has an intact *tad *secretion system. 35000HPΔ*flp1-3 *was impaired in its ability to form microcolonies and to attach to HFF in vitro when compared to its parent (35000HP). Complementation of the mutant with *flp1-3 *in trans restored the parental phenotype. To test whether expression of Flp1-3 was necessary for virulence in humans, ten healthy adult volunteers were experimentally infected with a fixed dose of 35000HP (ranging from 54 to 67 CFU) on one arm and three doses of 35000HPΔ*flp1-3 *(ranging from 63 to 961 CFU) on the other arm. The overall papule formation rate for the parent was 80% (95% confidence interval, CI, 55.2%-99.9%) and for the mutant was 70.0% (95% CI, 50.5%-89.5%) (*P *= 0.52). Mutant papules were significantly smaller (mean, 11.2 mm^2^) than were parent papules (21.8 mm^2^) 24 h after inoculation (*P *= 0.018). The overall pustule formation rates were 46.7% (95% CI 23.7-69.7%) at 30 parent sites and 6.7% (95% CI, 0.1-19.1%) at 30 mutant sites (*P *= 0.001).

**Conclusion:**

These data suggest that production and secretion of the Flp proteins contributes to microcolony formation and attachment to HFF cells in vitro. Expression of *flp1-3 *is also necessary for *H. ducreyi *to initiate disease and progress to pustule formation in humans. Future studies will focus on how Flp proteins contribute to microcolony formation and attachment in vivo.

## Background

The *tad *(tight adherence) locus is present in many Gram-positive and Gram-negative bacteria as well as the Archaea and likely represents an ancient subtype of type IV secretion systems that was horizontally transferred to many bacterial species early in the course of evolution. The *tad *genes are located on a mobile genomic island coined "the widespread colonization island" by Figurski and coworkers [1]. The functions of the *tad *locus gene products have been best described for *Aggregatibacter *(formerly *Actinobacillus*) *actinomycetemcomitans*, where they are essential for adherence, biofilm formation, and pathogenesis. The Tad proteins are predicted to form a macromolecular transport system for the assembly and secretion of fimbria or pili, which mediate adherence of the organism to different surfaces [2].

*Haemophilus ducreyi *is a gram-negative coccobacillus that causes the sexually transmitted genital ulcer disease chancroid, which is a public health concern because it increases the risk of transmission and acquisition of human immunodeficiency virus-1. *H. ducreyi *contains a 12.8 kb *flp *(fimbria like protein) operon with 15 genes (*flp1-flp2-flp3-orfBC-rcpAB-orfD-tadABCDEFG*) that encode products with homology to proteins encoded by the *tad *locus in *A. actinomycetemcomitans *[3,4]. *H. ducreyi *mutants that lack expression of either Flp1 and Flp2, which encode fimbria like proteins, or *tadA*, which has homology to NTPases of type IV secretion systems, have decreased abilities to attach to human foreskin fibroblasts (HFF) and to form microcolonies on HFF [4,5]. A mutant deficient in the expression of *tadA *was significantly but partially attenuated in the temperature dependent rabbit model of experimental chancroid, while a mutant lacking *flp1 *and *flp2 *expression was fully virulent in this model [4,5]. Thus, whether Flp-Tad-mediated adherence and/or microcolony formation are critical factors in the virulence of *H. ducreyi *is unclear [6].

In experimental and natural infection in humans, *H. ducreyi *forms aggregates, the first step in microcolony formation, and colocalizes with polymorphonuclear leukocytes and macrophages, which fail to ingest the organism. In human inoculation experiments, a *tadA *mutant is highly attenuated for virulence; whether the observed attenuation is due to the lack of secretion of the Flp proteins or other unidentified effectors by the *tad *locus is unclear [5]. Given the discrepancy in virulence between the *tadA *mutant and the *flp1flp2 *mutant in the temperature dependent rabbit model [5], here we constructed and characterized a *flp1-3 *deletion mutant. We tested the *flp1-3 *mutant for its ability to cause disease in human volunteers and its ability to form microcolonies and adhere to human fibroblasts. Our data indicate that expression of Flps is required for virulence and that Flp-Tad mediated adherence correlates with the virulence of *H. ducreyi *in humans. To our knowledge, this study is the first to provide definitive proof that expression of the Flp proteins is required for the virulence of a bacterial pathogen in humans.

## Results

### Construction and characterization of 35000HPΔflp1-3

An unmarked, in frame deletion mutant of the *flp1, flp2*, and *flp3 *genes was constructed in 35000HP using recombineering technology and designated 35000HPΔ*flp1-3 *[7,8]. Sequence analysis of 35000HPΔ*flp1-3 *confirmed that *flp1, flp2 *and *flp3 *had been replaced by a short ORF that consisted of the upstream region of *flp1*, the start codon of *flp1*, 81 bp encoding a scar peptide, and the last 21 bp of *flp3*, including its stop codon. By qRT-PCR, the expression levels of *tadA *and *tadG*, two genes downstream of *flp3*, were similar in 35000HPΔ*flp1-3 *compared to 35000HP (data not shown), suggesting that the remainder of the *flp *operon was normally transcribed. 35000HP and 35000HPΔ*flp1-3 *demonstrated identical growth rates in broth (data not shown). The LOS profiles and OMP patterns as analyzed by SDS-PAGE were similar for the mutant and the parent (data not shown).

### Human inoculation experiments

To determine whether the Flp proteins play a role in pathogenesis, 35000HPΔ*flp1-3 *was compared with 35000HP for virulence using a mutant parent comparison trial in the human model of infection. Ten healthy adults (six males, four females; 5 Caucasian, 5 black; age range 32 to 59; mean age ± standard deviation, 48 ± 9 years) volunteered for the study. Three subjects (volunteers 333, 334, and 335) were inoculated in the first iteration, three subjects (volunteers 336, 337, and 338) in the second iteration, one subject (volunteer 341) in the third iteration and three subjects (volunteers 342, 343, and 344) in the fourth iteration. In the first iteration, each subject was infected with a fixed EDD (61 CFU) of 35000HP at three sites on one arm and varying EDDs (63, 125, and 249 CFU) of 35000HPΔ*flp1-3 *on the other arm (Table 1). Since the ability of the mutant to form pustules seemed impaired (Table 1), we increased the dose of the mutant in subsequent iterations, as per protocol. In the second iteration, volunteers were inoculated with 65 CFU of the parent and 66, 131, and 262 CFU of the mutant (Table 1). In the third iteration, the volunteer was infected with 67 CFU of 35000HP and 240, 480, and 961 CFU of the mutant (Table 1). In the fourth iteration, volunteers were inoculated with 54 CFU of the parent and 104, 208 and 415 CFU of the mutant (Table 1).

**Table 1:** Response to inoculation of live *H.ducreyi *strains

Volunteer	Gender^a^	Days of Observation	Isolate^b^	Dose (cfu)	No. of Initial Papules	No. of Pustules
333	F	14	P	61	3	1

			M	63-249	2	0

334	M	9	P	61	3	0

			M	63-249	2	0

335	M	7	P	61	3	1

			M	63-249	3	0

336	F	6	P	65	0	0

			M	66-261	3	0

337	M	7	P	65	0	0

			M	66-261	1	0

338	F	8	P	65	3	2

			M	66-261	0	0

341	M	8	P	67	3	2

			M	240-961	3	2^c^

342	F	7	P	54	3	3

			M	104-415	3	0

343	M	6	P	54	3	3

			M	104-415	2	0

344	M	7	P	54	3	2

			M	104-415	2	0

a, F = female, M = male; b, P = parent, M = mutant

The overall papule formation rate for the parent was 80% (95% confidence interval, CI, 55.2%-99.9%) at 30 sites and for the mutant was 70.0% (95% CI, 50.5%-89.5%) at 30 sites (*P *= 0.52). Mutant papules were significantly smaller (mean, 11.2 mm^2^) than were parent papules (21.8 mm^2^) 24 h after inoculation (*P *= 0.018). The overall pustule formation rates were 46.7% (95% CI 23.7-69.7%) at 30 parent sites and 6.7% (95% CI, 0.1-19.1%) at 30 mutant sites (*P *= 0.001). Mutant pustules formed at only two sites in one volunteer. These results indicate that expression of one or more of the *flp1, flp2*, and *flp3 *genes in the context of the intact secretion/assembly complex is necessary for *H. ducreyi *to initiate disease and progress to pustule formation in humans.

*H. ducreyi *was recovered intermittently from surface cultures. Of the 30 sites that were inoculated with the parent, 11 (36.7%) yielded at least one positive surface culture, while 3 of 30 mutant sites (10.0%) yielded a positive surface culture (*P *= 0.019). All colonies recovered from sites inoculated with the parent (n = 626) or the mutant (n = 39) and colonies from the parent (n = 142) and mutant (n = 143) inocula were tested for the presence of *flp1-flp2-flp3 *and *fgbA *sequences by colony hybridization. The *fgbA *probe hybridized to all the colonies, while the *flp1-2-3 *probe hybridized only to the colonies obtained from the parent inoculated sites or the parent inocula. Thus, there was no cross contamination of mutant and parent sites during the course of the trial.

### *Deletion of *flp1flp2flp3 *affects attachment of 35000HP to HFF cells and microcolony formation*

**Figure 1:**
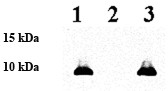
**Western Blot analysis of Flp1 and Flp2 expression by wild type, mutant, and complemented *H. ducreyi *strains**. Whole-cell lysates were probed with polyclonal rabbit Flp1 antiserum as the primary antibody. Lanes: 1, wild-type 35000HP(pLSSK); 2, 35000HPΔ*flp1-3*(pLSSK); 3, 35000HPΔ*flp1-3*(pJW1). Molecular markers are shown on the left.

To confirm the phenotype of 35000HPΔ*flp1-3*, we complemented 35000HPΔ*flp1-3 *in trans by electroporating it with plasmid pJW1, which encodes the Flp1, Flp2 and Flp3. *flp1, flp2, and flp3 *encode proteins with predicted molecular weights of 9.3 kDa, 8.9 kDa, and 9.9 kDa, respectively. Western blot analysis with a polyclonal sera that binds to Flp1 and Flp2 confirmed that 35000HPΔ*flp1-3*(pLSSK) lacked the ability to express the Flp1 and Flp2 proteins (Figure 1, lane 2) compared to 35000HP(pLSSK) (Figure 1, lane 1). Complementation of 35000HPΔ*flp1-3 *with plasmid pJW1 resulted in restoration of the expression of the Flp1 and Flp2 proteins as determined by Western blot (Figure 1, lane 3).

**Figure 2:**
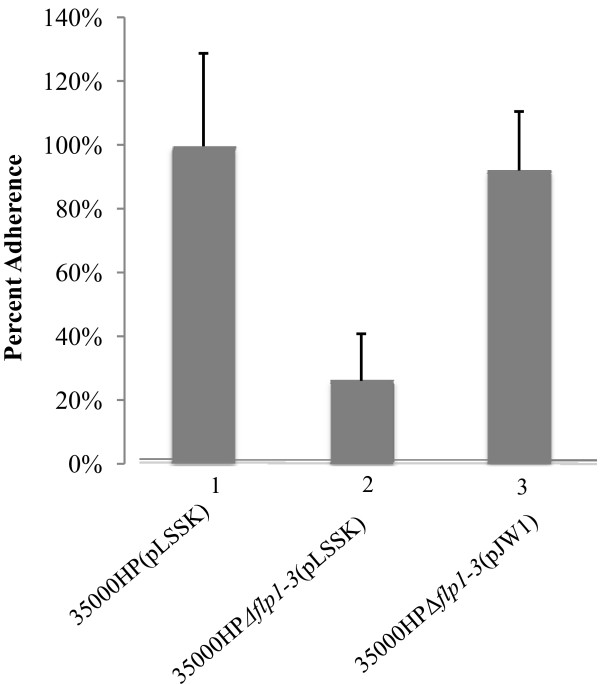
**Quantitative measurement of the binding of wild type, mutant, and complemented *H. ducreyi *strains to HFF cells**. Assays were performed as described in Materials and Methods. The data represented are a composite of five separate experiments. Bars: 1, wild-type 35000HP(pLSSK); 2, 35000HPΔ*flp1-3*(pLSSK); 3, 35000HPΔ*flp1-3*(pJW1).

35000HP(pLSSK), 35000HPΔ*flp1-3*(pLSSK), and 35000HPΔ*flp1-3*(pJW1) were also tested for their abilities to bind confluent HFF monolayers. 35000HPΔ*flp1-3*(pLSSK) significantly attached to HFF cells at lower levels (geomean ± standard deviation, 26.0% ± 15.0%) than did 35000HP(pLSSK) (100% ± 29.0%) (*P *= 0.018) (Figure 2). 35000HPΔ*flp1-3*(pJW1) adhered to HFF cells (92.0% ± 18.0%) at significantly higher levels than 35000HPΔ*flp1-3*(pLSSK) (*P *= 0.010) and at similar levels as 35000HP(pLSSK) (*P *= 0.32) (Figure 2).

**Figure 3:**
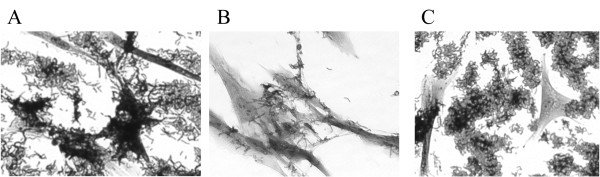
**Microcolony formation by (A) wild type 35000HP(pLSSK), (B) *flp1-3 *mutant 35000HPΔ*flp1-3*(pLSSK), and (C) complemented *flp1-3 *mutant 35000HPΔ*flp1-3 *(pJW1)**. Magnification ×400.

35000HP(pLSSK), 35000HPΔ*flp1-3*(pLSSK), and 35000HPΔ*flp1-3*(pJW1) were also compared for their abilities to form microcolonies after 24 h incubation with confluent HFF monolayers. 35000HP formed numerous, densely populated microcolonies on the surfaces of HFF cells [4] (Figure 3A). 35000HPΔ*flp1-3*(pLSSK) formed sparse and very small microcolonies (Figure 3B) when compared to 35000HP; the complemented mutant demonstrated a restored phenotype similar to 35000HP(pLSSK) (Figure 3C). Thus, complementation of the mutant restored the parental phenotypes.

## Discussion

For this study, we focused on whether the expression of the Flp proteins was necessary for virulence of *H. ducreyi*. We constructed an unmarked, in frame deletion mutant lacking the *flp1flp2flp3 *genes in 35000HP using a recombineering strategy [8,9] and found that 35000HPΔ*flp1-3 *was significantly impaired in its ability to cause disease in the human model of infection. *flp1-3 *joins *hgbA, dsrA, ncaA, lspA1-lspA2, pal, tadA sapBC *and *cpxA *as the ninth gene required for full virulence by *H. ducreyi *in the human inoculation experiments [9,10] (unpublished observations).

The *flp-tad *gene cluster is constitutively transcribed as a single polycistronic operon in vitro [4]. Relative to its expression during in vitro growth, *tadA *transcripts are enriched in experimental pustules, suggesting that the *flp-tad *operon is upregulated in vivo [11]. CpxRA is the only obvious intact two-component signal transduction system contained in *H. ducreyi*. Transcription of *flp1-3 *and several other major virulence determinants are negatively regulated by conditions that favor phosphorylation of CpxR [9,12,13]. Purified recombinant CpxR interacts with the promoter regions of the *flp *operon in electrophoretic mobility shift assays [13]. Deletion of *cpxA *leads to loss of CpxA phosphatase activity, activates CpxR, and cripples the ability of *H. ducreyi *to infect humans [9]. In contrast, a *cpxR *deletion mutant has no effect on or upregulates the expression of virulence determinants and is fully virulent in human volunteers [13]. Taken together, the data suggest that the *flp-tad *operon may be upregulated in vivo due to downregulation of CpxRA.

The human inoculation experiments are limited in that we are precluded by several regulatory bodies from testing *trans*-complemented mutants in humans. However, complementation of 35000HPΔ*flp1-3 *in trans restored the ability of the mutant to form microcolonies and bind to HFF cells, suggesting that the phenotype of the mutant is due to the deletion of the *flp *genes. In the human inoculation experiments, we use 35000HP to examine the role of virulence factors in *H. ducreyi *pathogenesis. There are two classes of *H. ducreyi *strains, which express different immunotypes and proteomes [14,15]. Although we were able to amplify *flp1-3 *alleles from six class I and three class II strains (data not shown), attempts to sequence the amplicons were unsuccessful, so we do not know if there is a difference in the *flp *genes in the class I and class II strains. 35000HP is a class I strain; whether the Flp proteins play a role in the virulence of class II strains is unknown.

We previously reported that a *tadA *mutant is attenuated for pustule formation in the human challenge model [5]. However, the *tadA *mutant, but not a *flp1flp2 *double mutant, is attenuated in the rabbit model of chancroid [4,5]. Nika et al previously reported that both the *flp1flp2 *mutant and the *tadA *mutant demonstrate decreased abilities to attach to HFF cells and form fewer microcolonies on HFF cells [4]. These data suggested that microcolony formation by itself is not a virulence factor for *H. ducreyi*. Although *H. ducreyi *does not appear to co-localize with fibroblasts in experimental or natural chancroid [16,17], our data indicate that adherence to HFF cells in vitro correlates with the virulence of *H. ducreyi *in humans. Similarly, both *flp1 *and *tadA *mutants fail to colonize or cause disease in a rat infection model with *A. actinomycetemcomitans *[1], and both *flp1 *mutant and *tadD *mutants of *Pasteurella multocida *are attenuated in the septicemic mouse model [18,19].

In *A. actinomycetemcomitans*, Flp pili are assembled as bundles of long fibers in which Flp1 is the major structural component [3,20]. However, there is no evidence that the Flp proteins are assembled into a pilus-like structure in *H. ducreyi *[4]. Several bacterial species including *A. actinomycetemcomitans *have two *flp *genes [2]. *H. ducreyi *contains three *flp *genes, which have between 50-80% similarity to one another [4]. Deletion of *flp1 *and *flp2 *results in decreased adherence of *H. ducreyi *to HFF cells and subsequent microcolony formation [4]; the function of Flp3 is unclear.

In vitro, *H. ducreyi *forms microcolonies, a key step in biofilm formation. In vivo, *H. ducreyi *forms aggregates and colocalizes with macrophages, PMNs, collagen and fibrin [16,17]. *H. ducreyi *contains a *luxS *homologue that has autoinducer (AI-2) activity in a *Vibrio harveyi*-based reporter system, and a *luxS *mutant is partially attenuated for virulence in human volunteers [21]. Taken together, these data suggest that the formation of microcolonies, aggregates and quorum sensing mechanisms may be important for *H. ducreyi *pathogenesis. Whether the Flp proteins contribute to this process by mediating attachment to host cells or initiating microcolony formation in the skin remains a subject for future investigation.

## Conclusions

We have constructed an unmarked, in frame deletion mutant lacking the *flp1flp2flp3 *genes in *H. ducreyi *strain 35000HP. The deletion mutant, 35000HPΔ*flp1-3*, has an intact *tad *secretion system. Our data show that production and secretion of the Flp proteins contributes to microcolony formation and attachment of 35000HP to HFF cells in vitro. Complementation of the mutant with *flp1-3 *in trans restored the parental phenotype. Additionally, expression of Flp1-3 is necessary for *H. ducreyi *to initiate disease and progress to pustule formation in humans. Future studies will focus on how Flp proteins contribute to microcolony formation and attachment in vivo.

## Methods

### Bacteria and culture conditions

35000HP is a human-passaged (HP) variant of strain 35000 and has been reported previously [22]. *H. ducreyi *strains were grown on chocolate agar plates supplemented with 1% IsoVitaleX at 33°C in 5% CO_2_. For the human inoculation experiments, *H. ducreyi *was grown in a protease peptone broth-based medium supplemented with 50 μg of hemin per ml, 1% IsoVitaleX and 5% heat-inactivated fetal calf serum (FCS) as described [23] or in a Columbia broth based medium with 2.5% heat-inactivated FCS for other experiments. When appropriate, the media were supplemented with chloramphenicol, spectinomycin, or kanamycin at 0.3 μg/ml, 200 μg/ml, or 20 μg/ml, respectively, to maintain plasmids or select for chromosomal integration of antibiotic resistance cassettes. *E. coli *strains were grown in Luria-Bertani broth or plates at 37°C, except for strain DY380, which was grown or maintained at 32°C and induced to express λ recombinase at 42°C.

### *Construction and characterization of a *flp1-3 *mutant of strain *35000HP

An unmarked, in frame deletion mutant of the *flp1, flp2*, and *flp3 *genes was made in *H. ducreyi *strain 35000HP using Flippase (FLP) recombinase technology as described previously [8,9]. Briefly, two 70 bp primers, P1 and P2, were designed for construction of a cassette (Table 2). The 3' end of each of these primers contained 20 bp complementary to regions 5' and 3' of a spectinomycin cassette flanked by FLP Recognition Target (FRT) sites in pRSM2832 [8]. The 5' portion of the P1 and P2 primers were homologous to regions 5' and 3' of *H. ducreyi flp1 *and *flp3*, respectively. PCR of pRSM2832 with P1 and P2 yielded a 2 Kb amplicon that contained the spectinomycin cassette flanked by FRT sites and 50 bp of DNA homologous to regions 5' and 3' of *H. ducreyi flp1 *and *flp3*, respectively. This amplicon was electroporated into *E. coli *DY380 harboring a cosmid size pBeloBAC clone containing the *flp *operon and flanking DNA. After induction of λ recombinase in this strain, spectinomycin-resistant clones were isolated. One clone was further characterized to demonstrate that the *flp1, -2 and -3 *genes were replaced with the spectinomycin cassette, with the exception of the *flp1 *start codon and the terminal 21 bp of the *flp3 *ORF. The construct was confirmed by sequence analysis.

**Table 2:** Primers used in this study

Primer	Sequence
P1	TAACCTAAAAAAACAACATAATTTATTTTATATTTGGAGAAAAAGATATGATTCCGGGGATCCGTCGACC

P2	GTATATATGGCACATATAAATTATGTGTTTTATAATCTACCTTTATTGAATGTAGGCTGGAGCTGCTTCG

P3	CGGTCACGATGGTTCAATGTCT

P4	AGCGTTTGACATCATCACCATACT

P5	TGCCTACAGCTCAAGTCACGTAA

P6	CCACTCGAAAGCGAAACTTGT

P7	CATCTCGAGCGCCACACTATCCAC

P8	CACTCTAGATTATAATCTACCTTT

P9	GGCTTAATTGCAGTCGCAGTTGCT

P10	GTGCAGCTTTACCTACTCCTCCTT

P11	ACTCCGCAGCTGATGCAATGAAAG

P12	CAAGCTTATCGATACCGTCGACCT

The pBeloBAC clone containing the insertion/deletion mutation in the *flp *genes was used as a template for PCR. The amplicon containing the insertionally inactivated *flp1flp2flp3 *genes and approximately 500 bp of flanking DNA 5' and 3' to the cassette was ligated into the suicide vector, pRSM2072, and then electroporated into 35000HP. Cointegrates were selected by growth on spectinomycin, then resolved by passage on plates containing spectinomycin and 5-bromo-4-chloro-3-indoly-β-D-galactopyranoside **(**X-Gal) [24]. Allelic exchange was confirmed by colony PCR.

To make an unmarked mutant, the plasmid, pRSM2975, which contains a temperature sensitive replicon, a kanamycin resistance cassette, and FLP recombinase under the control of the *tet *repressor, was transformed into the mutant [9]. Transformants were selected and maintained at 32°C on chocolate agar containing kanamycin. The FLP recombinase was induced to catalyze excision of the spectinomycin cassette resulting in a short unmarked ORF in place of the *flp1, flp2 *and *flp3 *genes and the plasmid was cured as described previously [9]. Colonies were replica plated on chocolate agar containing no antibiotics, 200 μg/ml of spectinomycin, and 20 μg/ml of kanamycin to identify clones that lost both the temperature sensitive plasmid and the spectinomycin cassette. The plasmid and the spectinomycin cassette were lost in 3/120 (2.5%) of the clones tested. One clone that had a deletion of the expected size by colony PCR was designated 35000HPΔ*flp1-3*.

Lipooligosaccharide (LOS) and outer membrane proteins (OMPs) were prepared from 35000HP and 35000HPΔ*flp1-3 *and were analyzed by sodium dodecyl sulfate-polyacrylamide gel electrophoresis, as described [25]. The growth of parent and mutant in broth cultures were also compared.

### RNA isolation and Real Time PCR

Bacterial RNA was prepared from mid-log phase organisms by using TRIzol Reagent (Invitrogen) according to manufacturer's instructions. After isolation, RNA was treated twice with DNaseI (Ambion) for 1 hour at 37°C and then purified by using the RNeasy system (Qiagen). Samples were checked by Agilent analysis. After optimizing primers so that their efficiencies were greater than 95%, we examined the level of transcript expression in RNA isolated from 35000HP and 35000HPΔ*flp1-3*. Using each bacterial RNA, and either the *tadA *primers (P3 and P4) (Table 2) or the *tadG *(P5 and P6) primers (Table 2) and SYBR Green, reactions were performed in triplicate using an ABI PRISM 7000 Sequence Detector (Applied Biosystems). Data were expressed as fold change of *tadA *and *tadG *in the mutant relative to the parent.

### Complementation of 35000HPΔflp1-3

To complement 35000HPΔ*flp1-3 *in trans, the *flp*1, *flp*2 and *flp*3 ORFs were amplified using the P7 primer with a *BamH1 *linker and the P8 primer with an *XhoI *linker. The resulting 1.58-kb amplicon was ligated into pCR-XL-TOPO (Invitrogen, Calsbad, Calf.). Transformants were selected on Luria-Bertani plates supplemented with kanamycin (50 μg/ml). The 1.58 kb insert was released from the vector by digestion with *Bam*HI and *Xho*I, ligated into pLSSK [26], and then transformed into *E. coli *DH5α. The plasmid was confirmed by restriction mapping and designated pJW1. *H. ducreyi *35000HPΔ*flp1-3 *was electroporated with pJW1. As controls, 35000HP and 35000HPΔ*flp1-3 *were electroporated with pLSSK. Transformants were selected on chocolate agar plates containing streptomycin (50 μ/ml) and transformants were saved and designated 35000HPΔ*flp1-3*(pJW1), 35000HP(pLSSK) and 35000HPΔ*flp1-3*(pLSSK).

### SDS-PAGE and Western Blot Analysis

Whole cell lysates were prepared from 35000HPΔ*flp1-3*(pJW1), 35000HPΔ*flp1-3*(pLSSK), and 35000HP(pLSSK) and subjected to SDS-PAGE as previously described [27]. In Western Blot analysis, whole cell lysates were probed with rabbit polyclonal sera that bind to Flp1 and Flp2 (kindly provided by Eric J. Hansen) as described elsewhere [4].

### Human inoculation protocol

Stocks of 35000HP and 35000HPΔ*flp1-3 *were prepared according to the US Food and Drug Administration guidelines (BB-IND 13046). For the human inoculation protocol, healthy adult male and female volunteers over 21 years of age were recruited for the study. Subjects gave informed consent for participation and for human immunodeficiency virus (HIV) serology, in accordance with the human experimentation guidelines of the U.S. Department of Health and Human Services and the institutional ethics committee of Indiana University-Purdue University of Indianapolis. The experimental protocol, preparation and inoculation of the bacteria, calculation of the estimated delivered dose (EDD), and clinical observations were all done exactly as described previously [10,28]. Subjects were observed until they reached clinical endpoint, which was defined as resolution of all sites, development of a pustule that was either painful or > 6 mm in diameter, or 14 days after inoculation. Subjects were then treated with one dose of oral ciprofloxacin as described [29]. Comparison of papule and pustule formation rates for the two strains were performed using a logistic regression model with generalized estimating equations (GEE) to account for the correlation among sites within the same individual, as previously described [28]. The GEE sandwich estimate for the standard errors was used to calculate 95% confidence intervals (95% CI) for these rates.

To confirm that the strains contained or lacked the *flp1flp2flp3 *genes, colonies from the inocula, surface cultures and biopsy specimens were replica plated and grown on nitrocellulose filters. Filters were probed with amplicons corresponding to either the *fgbA *(fibrinogen binder A) gene or the deleted portion of the *flp1flp2flp3 *genes. The *flp1flp2flp3 *and the *fgbA *probes were made using primers P9 and P10 and primers P11 and P12, respectively. Probes were labeled with digoxigenin using the DIG DNA Labeling Kit (Roche Applied Sciences, Penzberg, German) and detected with the DIG Easy Hyb protocol (Roche Applied Sciences) according to the manufacturer's instructions.

### Adherence assays

Adherence of bacteria to HFF was measured quantitatively as described previously [4]. Briefly, 24-well tissue culture plates (Costar, Corning, N.Y.) were inoculated with 10^5 ^HFF/well and grown to confluence. 35000HPΔ*flp1-3*(pJW1), 35000HPΔ*flp1-3*(pLSSK), and 35000HP(pLSSK) were grown in Columbia broth to an OD_660 _between 0.4 and 0.6 and harvested by centrifugation. Bacterial pellets were suspended using HFF medium and approximately 10^6 ^CFU were added to individual wells containing confluent HFF, centrifuged at 500 × *g*, and incubated for 2 h at 33°C. After nonadherent bacteria were removed by washing three times with HFF medium, 1 ml of trypsin-EDTA (Invitrogen) solution was added to each well and the plate was incubated for 5 min to liberate the bound bacteria. Serial dilutions of well contents were plated to quantitate HFF-bound bacteria. Percent adherence was calculated as the ratio of HFF-bound bacteria to initial CFU added per well. A paired Student's t test was used for the comparison of strains' abilities to attach to HFF cells.

### Microcolony formation assays

Confluent HFF monolayers were prepared and 35000HP(pLSSK), 35000HPΔ*flp1-3*(pLSSK), and 35000HPΔ*flp1-3*(pJW1) were grown as described for the adhesion assays. Bacterial pellets were resuspended in HFF medium to an OD_660 _of 0.1. Approximately 10^6 ^CFU for each strain was added to individual wells of confluent HFF cells, centrifuged at 500 × *g *for 4 min, and incubated for 24 h at 33°C. Wells were washed three times with 1 ml HFF medium and then stained with crystal violet [0.25% (wt/vol) crystal violet, 20% (vol/vol) methanol, 0.9% (wt/vol) NaCl, 0.02 M Tris-HCl (pH 7.5)] for 20 min at room temperature.

## Potential conflicts of Interest

The authors have no conflicts of interest to report.

## Authors' contributions

SAC carried out the adherence and microcolony formation assays. JW constructed and characterized the complemented mutant. BB and RSM constructed and characterized the *flp1-3 *deletion mutant. KRF prepared the bacterial strains used for the human inoculation experiments and participated in the mutant characterization. BWZ and SE prepared the regulatory documents and performed the clinical observations for the human inoculation experiments. BPK performed the statistical analysis. DMJ and RSM participated in the design of the study and drafted the manuscript. All authors read and approved the final manuscript.
